# GlobSnow v3.0 Northern Hemisphere snow water equivalent dataset

**DOI:** 10.1038/s41597-021-00939-2

**Published:** 2021-07-01

**Authors:** Kari Luojus, Jouni Pulliainen, Matias Takala, Juha Lemmetyinen, Colleen Mortimer, Chris Derksen, Lawrence Mudryk, Mikko Moisander, Mwaba Hiltunen, Tuomo Smolander, Jaakko Ikonen, Juval Cohen, Miia Salminen, Johannes Norberg, Katriina Veijola, Pinja Venäläinen

**Affiliations:** 1 Finnish Meteorological Institute, PO Box 503, FIN-00101 Helsinki, Finland; 2 Climate Research Divison, Environment and Climate Change Canada, 4905 Dufferin Street Toronto, Ontario, M3H 5T4 Canada

**Keywords:** Hydrology, Cryospheric science, Cryospheric science, Water resources

## Abstract

We describe the Northern Hemisphere terrestrial snow water equivalent (SWE) time series covering 1979–2018, containing daily, monthly and monthly bias-corrected SWE estimates. The GlobSnow v3.0 SWE dataset combines satellite-based passive microwave radiometer data (Nimbus-7 SMMR, DMSP SSM/I and DMSP SSMIS) with ground based synoptic snow depth observations using bayesian data assimilation, incorporating the HUT Snow Emission model. The original GlobSnow SWE retrieval methodology has been further developed and is presented in its current form in this publication. The described GlobSnow v3.0 monthly bias-corrected dataset was applied to provide continental scale estimates on the annual maximum snow mass and its trend during the period 1980 to 2018.

## Abstract (summary)

**Table:** 

Measurement(s)	snow • snow mass • snow water equivalent
Technology Type(s)	satellite imaging
Sample Characteristic - Environment	cryosphere
Sample Characteristic - Location	Northern Hemisphere

Machine-accessible metadata file describing the reported data: 10.6084/m9.figshare.14336612

## Background & Summary

The approach for generating the GlobSnow v3.0 (GSv3) snow water equivalent (SWE) daily, monthly and monthly bias-corrected datasets is described. The bias-corrected monthly dataset was used to produce a well-constrained reconstruction of the Northern Hemisphere snow mass and its trends for 1980–2018^1^.

The European Space Agency (ESA) GlobSnow project, and successive product development within the ESA Climate Change Initiative, have produced a family of daily hemisphere-scale satellite-based SWE data records spanning over 40 years. The most recent GSv3 SWE data record, based on GlobSnow methodology^2,3^ has been further refined and now incorporates a novel bias-correction procedure^1^.

The retrieval methodology combines satellite-based passive microwave (PMW) measurements with ground-based synoptic weather station observations by Bayesian non-linear iterative assimilation. A background snow-depth (SD) field from re-gridded surface SD observations and a passive microwave emission model^4,5^ are key components of the retrieval scheme. Due to the importance of the weather station SD measurements on the SWE retrieval, the GSv3 dataset contains improved screening for consistency of the applied SD observations through the time series.

The data record is based on 19 and 37 GHz measurements from the Scanning Multichannel Microwave Radiometer (SMMR) onboard NIMBUS-7, and Special Sensor Microwave/Imager (SSM/I) and Special Sensor Microwave Imager/Sounder (SSMIS) instruments onboard the Defense Meteorological Satellite Program (DMSP) F-series satellites. These frequencies have original spatial resolutions between 15 and 69 km, but are re-gridded to 25 × 25 km pixel spacing in the Equal-Area Scalable Earth (EASE) Grid north azimuthal equal-area projection^6^. The SWE retrievals are produced daily for non-alpine regions of the Northern Hemisphere. Known limitations in alpine terrain are related to the coarse resolution of the satellite passive microwave measurements, and sparse surface observations which are unable to capture the high spatial variability in SD. A complex-terrain mask is therefore applied based on the sub-grid variability in elevation determined from a digital elevation model (standard deviation of elevation within a grid cell exceeding 200 m). All land ice and large lakes (grid cells with >50% water fraction) are also masked; retrievals are not produced for coastal regions of Greenland.

The daily SWE record is averaged to the monthly scale, and a novel bias-correction procedure is subsequently applied to the monthly time series, to mitigate a low bias in the retrievals under deep snow (SWE >150 mm) conditions. The bias-correction is based on analysis of transect SWE measurements (‘snow courses’) which are independent from the weather station SD observations.

A detailed performance assessment is presented for the GSv3 dataset whereby the GSv3 SWE retrievals were matched with co-located snow transect SWE observations for 1980–2016 and bias, root-mean-square error (RMSE), and correlation statistics were computed. The assessment was made for all samples and for a subset consisting only of shallow to moderate snow conditions (SWE <150 mm). The overall RMSE for 1980–2016 is 52.6 mm. GSv3 slightly overestimates SWE in Eurasia and underestimates across Canada where mean SWE at the snow transect locations is considerably greater. The overall RMSE for shallow to moderate snow (SWE <150 mm) is 32.7 mm. There is no apparent trend in bias, RMSE, or correlation over the 1980–2018 SWE time series.

The GSv3 SWE retrieval performance is presented for different seasons and land cover categories, with the effect of the bias correction further analyzed. The performance of the GSv3 SWE retrieval versus the direct interpolation of *in situ* weather station SD data (where SWE is obtained solely from the SD observations and no PMW data is assimilated) is also assessed and presented.

The GSv3 product with a spatial bias correction has been applied to provide continental scale estimates of the annual maximum snow mass (excluding mountain snow) and its trend across the period from 1980 to 2018^1^. The results show that, on average, the annual maximum snow mass was 3,062 ± 35 gigatonnes for that period. For North America the bias-corrected GSv3 estimate was 1,128 ± 31 gigatonnes and 1,934 ± 35 Gt for Eurasia. These estimates were obtained for land regions above 40°N, excluding mountain areas. The monthly snow mass estimates show that the March values correspond to peak snow mass. In addition to refining the snow mass climatology, continental and regional scale trends of monthly snow mass were analyzed and they indicated that for March, the 1980–2018 trend in Eurasia is negligible, whereas it is significantly negative for North America (−46 ± 42 gigatonnes per decade)^1^. Spatially, there is high regional variability in GSv3 snow mass trends on both continents.

## Methods

### Overview of the SWE Retrieval method

The SWE processing system relies on Bayesian assimilation which combines ground-based data with satellite-borne observations^2^. The method applies two vertically polarized satellite-based brightness temperature observations at 19 and 37 GHz and a scene brightness temperature model (the HUT snow emission model^4^). First, snow microstructure described by an ‘effective snow grain size’ is estimated for grid cells with a coincident weather station SD observation. Effective snow grain size is used in the HUT model as a scalable model input parameter to optimize agreement with the satellite measurements. These values of grain size are used to interpolate a background map of the effective grain size, including an estimate of the effective grain size error. This spatially continuous map of grain size is then used as an input for a second HUT model inversion to provide an estimate of SWE. In the inversion process, the effective grain size in each grid cell is weighed with its respective error estimate and a constant value of snow density is applied. The spatially continuous SWE map obtained from the second run of the HUT snow model described above is fused with a background SD field (converted to SWE using 0.24 g cm^−3^) to obtain a final estimate of SWE using a Bayesian non-linear iterative assimilation approach (which weights the information sources with their estimated variances). The background SD field is generated from the same weather station SD observations used to estimate the effective snow grain size using kriging interpolation methods.

The microwave scattering response to SWE saturates under deep snow conditions (>150 mm) and model inversion of SD/SWE over areas of wet snow is not feasible because the microwave signal is absorbed rather than scattered. For these reasons, the method decreases the weight of satellite data for deep dry snowpacks and wet snow by assessing the modeled sensitivity of brightness temperature to SWE within the data assimilation procedure^2,3^.

Before SWE retrieval, dry snow is identified from brightness temperature data^7^. For the autumn snow accumulation season (August to December), the dry snow detection is used to construct a cumulative snow presence mask to track the advance of snow extent (SWE estimates are restricted to the domain indicated by the cumulative snow presence mask). During spring the overall mapped snow extent is determined from the cumulative mask, which (as the melt season proceeds) is reduced using a satellite passive microwave derived estimate for the end of snow melt season for each grid cell^8^.

The snow part of the applied scene brightness temperature model is based on the semi-empirical HUT snow emission model which describes the brightness temperature from a multi-layer snowpack covering frozen ground in the frequency range of 11 to 94 GHz^4,5^. Input parameters to the model include snowpack depth, density, effective grain size, snow volumetric moisture and temperature. Separate modules account for ground emission and the effect of vegetation and atmosphere. Comparisons of HUT model simulations to airborne and tower-based observations, reported elsewhere (e.g.^9,10^), demonstrate the ability of the model to simulate different snow conditions and land cover regimes. Intercomparisons with other emission models show comparable performance when driven by *in situ* data^11,12^ or physical model outputs^13^, although the HUT model has the tendency to underestimate brightness temperatures for deep snowpacks^12^.

#### Basic underlying assumptions

Passive microwave sensitivity to SWE is based on the attenuating effect of snow cover on the naturally emitted brightness temperature from the ground surface. The ground brightness temperature is scattered and absorbed by the overlying snow medium, typically resulting in a decreasing brightness temperature with increasing (dry) snow mass. The scattering intensity increases as the wavelength approaches the size of the scattering particles. Considering that individual snow particles tend to range from 0.5 to 4 mm in the long axis direction, high microwave frequencies (short wavelengths) will be scattered more than low frequencies (long wavelengths). The intensity of absorption can be related to the dielectric properties of snow, with snow density largely defining the permittivity for dry snow. Absorption at microwave frequencies increases dramatically with the inclusion of free water (moisture) in snow, resulting in distinct differences of microwave signatures from dry and wet snowpacks.

Initial investigations pointed out the sensitivity of microwave emission from snowpacks to the total snow water equivalent^14^. This led to the development of various retrieval approaches of SWE from the earliest passive microwave instruments in space (e.g.^15,16^). From the available set of observed frequencies, most SWE algorithms employ the ~37 GHz and ~19 GHz channels in combination. These two frequencies are available continuously since 1979. The scattering from snow at 19 GHz is smaller when compared to 37 GHz, while the emissivity of frozen soil and snow is estimated to be largely similar at both frequencies. The brightness temperature difference of the two channels can be related to snow depth (or SWE), with the additional benefit that the effect of variations in physical temperature on the measured brightness temperature are reduced (relative to the analysis of single frequencies). Similarly, observing a channel difference reduces or even cancels out systematic errors of the observation, provided that the errors in the two observations are similar (e.g. due to using common calibration targets on a space-borne sensor). Typically, the vertically polarized channel at 19 and 37 GHz is preferred due to the inherent decreased sensitivity to snow layering (e.g.^17^).

A basic assumption in the data assimilation procedure that combines spaceborne passive microwave observations and synoptic weather station data to estimate snow depth is that the background snow depth field, interpolated from weather station data, provides meaningful information on the spatial patterns of snow depth. A limitation of the methodology is that this assumption does not hold for complex terrain (mountains). Further, the methodology is not suitable for snow cover on top of ice sheets, sea ice or glaciers.

#### Input data

The main input data are synoptic snow depth (SD) observations and spaceborne passive microwave brightness temperatures from the Scanning Multichannel Microwave Radiometer (SMMR), Special Sensor Microwave/Imager (SSM/I) and Special Sensor Microwave Imager/Sounder (SSMIS) data from Nimbus-7 and DMSP F-series satellites. The most important frequencies for SWE retrieval and snow detection are 19 GHz (reference measurement with very little scattering from the snow volume) and 37 GHz (sensitive to volume scattering by dry snow), which are available in all instruments. The satellite datasets are described in detail in Data Records section.

Ground-based SD data were acquired from the Finnish Meteorological Institute (FMI) weather station observation database, augmented from several archive sources, including the European Centre for Medium-Range Weather Forecasts (ECMWF), The United States National Climatic Data Centre (NCDC), The All-Russia Research Institute of Hydrometeorological Information-World Data Centre (RIHMI-WDC) and The Meteorological Service of Canada (MSC) archives, as described in the Data Records section.

In the assimilation of SD values with space-borne estimates, a density value of 0.24 g cm^−3^ is assumed in estimating SWE. In the assimilation procedure the spatial small-scale variability of SD is considered by assigning a variance of 150 cm^2^ to the weather station observations over forested areas, and a variance of 400 cm^2^ for open areas. These variance estimates describe how well a single-point SD observation describes the snow depth over a larger area surrounding the measurement site, and were determined from available FMI, Finnish Environment Institute (SYKE) and Environment and Climate Change Canada (ECCC) snow transect measurements, as well as experimental field campaign data from across Finland and Canada.

Daily SD background fields were generated from observations at synoptic weather station locations acquired from multiple archives for the years 1979–2018. For each measurement, the exact location, date of measurement, and SD are required. The long-term weather station data is pre-processed before utilization in the SWE retrieval to remove outliers and improve the overall consistency of the data, as described in the Methods section.

Land use and, most importantly, forest cover fraction are derived from ESA GlobCover 2009 300 m data^18^. Stem volume is required as an input parameter to the emission model to compensate for forest cover effects^4,19^; average stem volumes are estimated by the ESA BIOMASAR^20^ data records as described in the Methods section.

The following auxiliary datasets are used to mask out water and complex terrain (mountain) pixels:

- ESA CCI Land Cover from 2000: water fraction is aggregated to the 25 km grid cell spacing of the SWE product, pixels with a water fraction >50% are masked as water.
- ETOPO5^21^: if the standard deviation of the elevation within a 25 km grid cell is above 200 m it is masked as complex terrain.

### The Forward model applied in SWE retrieval

#### Calculation of brightness temperature for a satellite scene

For a satellite scene consisting of a mixture of non-forested terrain, forests, and snow-covered lake ice, the bottom-of-atmosphere brightness temperature T_B,BOA_ is calculated so that:

1 $${T}_{B,BOA}=\left(1-FF-LF\right){T}_{B,snow}+FF\cdot {T}_{B,forest}+LF\cdot {T}_{B,lake}$$

where *FF* is the forest fraction and *LF* the lake fraction of a given grid cell. ${T}_{B,snow}$, ${T}_{B,forest}$, and ${T}_{B,lake}$ are the brightness temperatures emitted from non-forested terrain (ground/snow), forested terrain, and lake ice, respectively. Land cover fractions *FF* and *LF* are determined from ESA GlobCover data resampled to the 25 km EASE grid. A statistical approach is used to calculate top-of-atmosphere brightness temperatures from *T*_B,BOA_, statistics are based on studies covering the Northern Hemisphere^4,22,23^.

#### Brightness temperature from snow-covered ground

The brightness temperature ${T}_{B,snow}$ for snow-covered, non-forested terrain is calculated using the HUT snow emission model^4^. The model is a radiative transfer-based, semi-empirical model which calculates the emission from a single homogenous snowpack. The current approach utilizes multi-layer modification which allows the simulation of brightness temperature from a stacked system of snow or ice layers^5^.

The absorption coefficient in the HUT model is determined from the complex dielectric constant of dry snow, applying the Polder-van Santen mixing model for the imaginary part^24^. The calculation of the dielectric constant for dry snow as well as effects of possible liquid water and salinity inclusions, are described through empirical formulae^25^. Emission from the snow layer is considered as both up- and down-welling emission. These are, in turn, reflected from interfaces between layers (air-snow, snow-ground). The transmission and multiple reflections between layer interfaces are calculated using the incoherent power transfer approach.

Applying the delta-Eddington approximation to the radiative transfer equation, the HUT model assumes that most of the scattered radiation in a snowpack is concentrated in the forward direction (of propagation) due to multiple scattering within the snow media, based on^26^, which assumes that losses due to scattering are approximately equal to generation of incoherent intensity by scattering. However the omission of the backward scattering component as well as omission of trapped radiation will lead to underestimation of brightness temperature for deep snowpacks^12^. In the HUT model, the rough bare soil reflectivity model^27^ is applied to simulate the upwelling brightness temperature of the soil medium.

#### Brightness temperature from forest vegetation

The brightness temperature over forested portions of the grid cell ${T}_{B,forest}$ is derived from ${T}_{B,snow}$ using a simple approximation so that:

2 $${T}_{B,forest}={t}_{veg}\cdot {T}_{B,snow}+\left(1-{t}_{veg}\right)\cdot {T}_{veg}+\left(1-{t}_{veg}\right)\cdot \left(1-{e}_{snow}\right)\cdot {t}_{veg}\cdot {T}_{veg}$$

where ${t}_{veg}$ is the one-way transmissivity of the forest vegetation layer, ${T}_{veg}$ the physical temperature of the vegetation (considered to be equal to air, snow and ground temperatures, ${T}_{veg}={T}_{air}={T}_{snow}={T}_{gnd}=-\,{5}^{^\circ }{\rm{C}}$) and ${e}_{snow}$ the emissivity of the snow covered ground system. The choice of −5 °C is based on experimental data^28^ and follows the previous publications^2–4^. Moreover the impact of physical temperature is minimal on the simulated brightness temperature difference of two frequencies applied in the retrieval (typically <1 K, and <3 K for extreme cases).

For the GSv3 SWE retrieval, the one-way forest transmissivity ${t}_{veg}$ is calculated by^19^;

3 $${t}_{veg}={e}^{-{\kappa }_{e}SV}$$

where ${{\rm{\kappa }}}_{e}$ is the forest vegetation extinction coefficient, and *SV* the forest stem volume (biomass). The coefficients were determined using airborne data^19^ for the key frequencies of 10.65, 18.7 and 36.5 GHz, and were validated for the range 0–100 m^3^ ha^−1^. The model^29^ applied in previous GlobSnow versions (1.0 and 2.0) saturated even for modest stem volumes (50 m^3^ ha^−1^ at 18.7 GHz and 100 m^3^ ha^−1^ at 36.5 GHz), which contradicts observational data. The earlier GlobSnow products also applied a constant stem volume of 80 m^3^ ha^−1^ for the entire Northern Hemisphere, due to lack of a reliable stem volume dataset at the time of development of those products.

Equation (3) and the extinction coefficients of ${{\rm{\kappa }}}_{e}$ = 0.007 at 18.7 GHz and ${{\rm{\kappa }}}_{e}$ = 0.011 at 36.5 GHz (for V-polarization) are now applied and replace the transmissivity model applied in the earlier SWE retrieval framework^29^. The ESA BIOMASAR^20^ stem volume estimates are applied to calculate spatially variable transmissivity, replacing the constant stem volume applied in earlier versions.

#### Brightness temperature from lake scenes

Earlier versions of the algorithm used in GlobSnow considered the brightness temperature over sub-grid lake fractions to be equal to that of snow covered ground. In GSv3, ${T}_{B,lake}$ is calculated separately using the multiple-layer version of the HUT snow emission model^5^, which considers frozen lakes as a stacked system of water, ice and snow. While introducing the cumulative effect of multiple reflections in a system of stacked layers, the original formulation of radiation scattering and absorption in individual snow layers was not altered. The mathematical solution^2^ was formulated for practical applications and is considered for scenes including lake ice in the GlobSnow SWE method following certain underlying generalizations^30^.

Based on a comparison of measurements of snow depth over lakes and over land in Finland^30^, the depth of the snow layer on ice in the forward simulation of ${T}_{B,lake}$ is considered to be always half of the equivalent snowpack on land. Snow density and grain size are considered to be identical. The depth (thickness) of the ice layer is considered to be a constant 50 cm, an approximation based on the mean maximum ice thickness over Finnish lakes in March^30,31^. The physical temperature of ice is considered to be equal to snow temperature (${T}_{ice}={T}_{snow}=-\,{5}^{^\circ }{\rm{C}}$), while the temperature of the underlying water layer is considered to be *T*_*water*_ = 0 °C.

### The SWE retrieval procedure

**Fig. 1:**
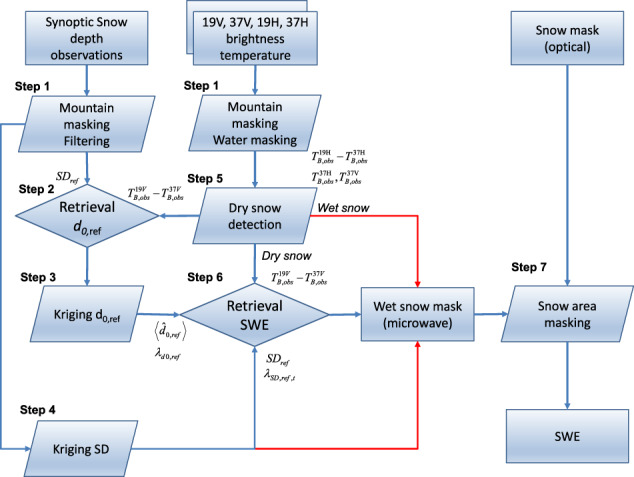
Processing chain for GSv3 SWE retrieval. For the wet snow regions, where satellite retrieval is not feasible, the SWE estimates are derived from the background SD field. Areas identified as snow-free using satellite-based optical and PMW data are masked out.

**Fig. 2:**
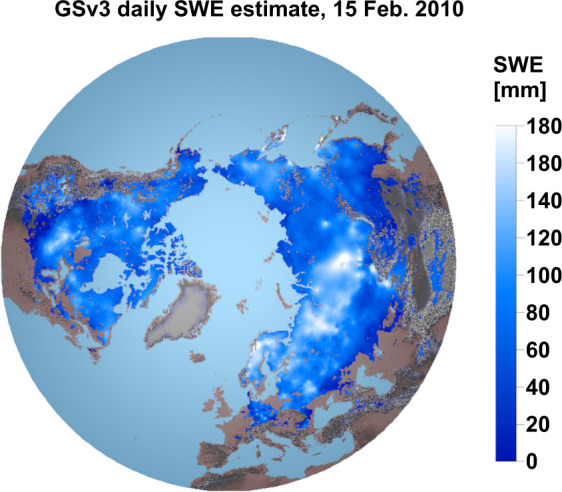
GlobSnow v3.0 (GSv3) daily SWE product for 15 February 2010.

The general processing chain for the GSv3 SWE product is given in Fig. 1 and explained in detail below. Northern Hemisphere GSv3 SWE map for 15 February 2010 is shown in Fig. 2, which shows that SWE estimates are not provided over sea ice, large lakes, glaciers, Greenland, nor complex terrain (mountains), as the retrieval is focused on terrestrial seasonal snow for which the PMW retrievals are feasible.

**Step 1**: The mountain mask and high SD value filter are applied to synoptic SD data. The high value mask removes the deepest 1.5% of reported snow depth values in order to avoid spurious or erroneous deep snow observations. The mountain mask criterion removes all observations that fall within EASE-grid cells with a height standard deviation above 200 m within the grid cell.

The mountain mask, water mask and dry snow masks (**Step 5**) are applied to satellite brightness temperature data. The water mask removes grid cells with over 50% water. The SWE retrieval from satellite data is performed for dry snow pixels. For wet snow, the final SWE estimates are based solely on the weather station kriged background SD field (assuming a constant snow density of 0.24 g cm^−3^ when converting SD to SWE).

**Step 2**: Numerical inversion of the multi-layer HUT snow emission model^2^ is performed for grid cells containing synoptic snow depth observations to retrieve values of effective grain size *d*_0_. The model is fit to spaceborne observed *T*_B_ values at the locations of weather stations by optimizing the value of snow grain size *d*_0_. The fitting procedure is:

4 $$\min {d}_{0}{\left\{\left({{\rm{T}}}_{{\rm{B}},19{\rm{V}},{\rm{mod}}}\left({d}_{0},S{D}_{gr}\right)-{{\rm{T}}}_{{\rm{B}},37{\rm{V}},{\rm{mod}}}\left({d}_{0},S{D}_{gr}\right)\right)-\left({{\rm{T}}}_{{\rm{B}},19{\rm{V}},obs}-{{\rm{T}}}_{{\rm{B}},37{\rm{V}},{\rm{obs}}}\right)\right\}}^{2}$$

where the known snow depth (on ground) is $S{D}_{gr}$, ${T}_{B,19V}$ and ${T}_{B,37V}$ denote the vertically polarized brightness temperature at approximately 19 and 37 GHz with indices *mod* and *obs* referring to modelled and observed values, respectively. Vertical polarization is used because it correlates best with SWE in the boreal forest zone^4,32^. Snow density is treated with a constant value of 0.24 g cm^−3^. At each synoptic SD station location, the final *d*_0,ref_ estimate (and its standard deviation *λ*) is obtained by averaging values obtained for the ensemble of the nearest stations, so that

5 $$\left\langle {\widehat{d}}_{0,ref}\right\rangle =\frac{1}{M}\mathop{\sum }\limits_{j=1}^{M}{\widehat{d}}_{0,ref,j}$$

6 $${\lambda }_{d0,ref}=\sqrt{\frac{1}{M-1}\mathop{\sum }\limits_{j=1}^{M}{\left({\widehat{d}}_{0,ref,j}-\left\langle {\widehat{d}}_{0,ref}\right\rangle \right)}^{2}}$$

where *M* is the number of stations (default 6).

**Step 3**: The effective grain size and its variance are interpolated over the full domain of brightness temperature observations (35° N to 85° N latitude and 180° W to 180° E longitude at a resolution of 25 km) using kriging interpolation, to obtain a spatially continuous field of effective grain size and its variance.

**Step 4**: The synoptic SD observations are interpolated over the same brightness temperature domain also using kriging interpolation. The variance λ_D_^2^ assigned to individual SD observations is set at 150 cm^2^ for open areas and 400 cm^2^ for forested areas. As an output, a spatially continuous estimate of SD and its variance are obtained.

**Step 5**: A dry snow mask is determined from the brightness temperature data to identify areas where dry snow is not present, and areas with wet snow cover. For dry snow, the following conditions^7^ need to be met:

7 $$\begin{array}{c}S{D}_{i}=15.9\cdot ({T}_{B,obs}^{19H}-{T}_{B,obs}^{37H}) > 80(mm)\\ {T}_{B,obs}^{37H} < 240K\\ {T}_{B,obs}^{37V} < 250K\end{array}$$

where *SD*_*i*_ is indicative snow depth for the given pixel, and needs to be above 80 (mm) and observed brightness temperatures of 37H and 37 V need to be below thresholds of 240 K and 250 K respectively for dry snow to be indicated. Only grid cells identified as dry snow are subject to the SWE retrieval process. Areas identified as wet snow for the given day are assigned a SWE value based on the kriging-interpolated SD map (from **Step 4**), converted to SWE. (wet snow masking is carried out on a daily basis, not cumulatively).

**Step 6**: Observed brightness temperatures, the effective grain size (**Step 2** and **3**) and effective grain size variance, over the whole area of interest are ingested into a numerical inversion of the HUT snow emission model to retrieve bulk SWE. Similar to the retrieval of effective grain size, an iterative cost function is applied. HUT snow emission model estimates are matched to observations numerically by fluctuating the SWE value. The background field of *SD*_*t*_ is applied to constrain the retrieval. A constant value of snow density (0.24 g cm^−3^) is applied to calculate *SWE*_*t*_ from *SD*_*t*_ where *t* refers to day in question. The cost function constrains the grain size value according to the predicted background grain size and the estimated variance produced in **Step 3**. Thus, assimilation adaptively weighs the space-borne brightness temperature observations and the background *SD*_*t*_ field (produced in **Step 4**) to estimate a final SWE and a measure of statistical uncertainty (in the form of a variance estimate) on a pixel basis:

8 $${\min }_{S{D}_{t}}\left\{{\left(\frac{\left({T}_{B,{\rm{mod}}}^{19V}\left(S{D}_{t}\right)-{T}_{B,{\rm{mod}}}^{37V}\left(S{D}_{t}\right)\right)-\left({T}_{B,obs}^{19V}-{T}_{B,obs}^{37V}\right)}{{\sigma }_{t}}\right)}^{2}+{\left(\frac{S{D}_{t}-S{\widehat{D}}_{ref,t}}{{\lambda }_{SD,ref,t}}\right)}^{2}\right\}$$

where $S{\widehat{D}}_{ref,t}$ is the snow depth estimate from the kriging interpolation for the day under consideration *t*. ${\lambda }_{SD,ref,t}$ is the estimate of standard deviation from the kriging interpolation, and *SD*_*t*_ is the snow depth for which Eq. (8) is minimized.

The variance of *T*_B_, σ_t_^2^, is estimated by approximating Δ*T*_*B*_ ($\Delta {T}_{B}={T}_{B}^{19}-{T}_{B}^{37}$) as function of snow depth and grain size in a Taylor series:

9 $$\Delta {T}_{B}\left({D}_{t},{d}_{0}\right)\approx \Delta {T}_{B}\left({D}_{t},{\widehat{d}}_{0,ref,t}\right)+\frac{\partial \Delta {T}_{B}\left({D}_{t},{\widehat{d}}_{0,ref,t}\right)}{\partial {d}_{0}}\left({d}_{0}-{\widehat{d}}_{0,ref,t}\right)$$

10 $${\sigma }_{t}^{2}={\rm{var}}\left(\Delta {T}_{B}\left({D}_{t},{\widehat{d}}_{0,ref,t}\right)\right)={\left(\frac{\partial \Delta {T}_{B}\left({D}_{t},{\widehat{d}}_{0,ref,t}\right)}{\partial {d}_{0}}\right)}^{2}{\lambda }_{d0,ref,t}^{2}$$

In practice, the variance σ_t_^2^ adjusts the weight of brightness temperature data with respect to the weight of the background SD field (parameter λ_D,ref_). A basic feature of the algorithm is that if the sensitivity of space-borne radiometer observations to SWE is assessed to be close to zero by formulas (9) and (10), the weight of radiometer data on the final SWE product approaches zero (this is the case if the magnitude of SWE is very high). The higher the estimated sensitivity of T_B_ to SWE, the higher is the weight given to the radiometer data. Thus, the weight of the radiometer data varies both temporally and spatially in order to provide a maximum likelihood estimate of SWE. The assimilation procedure is performed daily for dry snow pixels that are not excluded by water and complex terrain (mountain) masks.

**Step 7**: Snow-free areas are detected and cleared (SWE = 0 mm, inserted) using a combination of radiometer-derived information and snow extent information from optical remote sensing. A time-series detection approach^8^ is used to estimate the end of snow-melt season and any remaining SWE estimates are cleared from those pixels. After this, SWE estimates are also cleared from regions where optical data indicate snow-free conditions. The Japan Aerospace Exploration Agency (JAXA) JASMES 5 km Snow Extent data product 1978–2018^33^ is used to construct a cumulative snow mask in 25 km EASE-Grid projection for the SWE product time span. Cumulative masking retains the latest cloud-free observation for each EASE-Grid pixel, and uses the daily product to update snow-free/snow-covered conditions, based on a 25% snow cover fraction threshold.

### Uncertainty estimation for SWE retrieval

The GSv3 SWE product contains pixel-wise information of the retrieved SWE along with an uncertainty estimate which represents the total product error (determined for each individual pixel and each day) composed of a statistical random error component and a systematic error component. The statistical component is derived using an error propagation analysis^34^ and the systematic error component was determined using the snow transect reference dataset.

The statistical error component is the theoretical error estimated through an adaptive formulation^35^ that takes into account both the spatially and temporally varying characteristics of the weather station snow depth observations and the spaceborne microwave radiometer measurements^2,3^.

The systematic error is defined as the sum of all other error factors not considered in the statistical error analysis. These include unknown error that vary spatially and fluctuate temporally such as the inaccuracy of forward modeling, which can result in biases to the outcome of inversion algorithms (geophysical retrievals). Such derived biases of snow retrieval algorithms can be different for different snow regimes and they may show variability between seasons and from one winter to another. Based on the above, the systematic error is calculated as the residual from the total (absolute) error and the statistical error, where the total error itself is determined through analysis with the independent snow transect validation data^35^.

### Generation of monthly SWE products and bias-correction

The monthly aggregated snow water equivalent product (Level B) is calculated from all available daily SWE estimates for the given calendar month. The number of available daily data (daily products are considered Level A) is less than the total number of days in some months due to missing satellite data. During the SMMR era (1979 to 1987) satellite PMW data are only available every other day. The variation of the available daily data is not accounted for in the monthly product, only a simple mathematical average is provided. For the months that have zero observed daily SWE products, there are no monthly SWE estimates (typically for the summer months).

Monthly SWE products can be bias-corrected by applying available independent snow course observations that cover the Northern Hemisphere^1^. The principle of the bias correction method is to investigate the difference between the mean SWE observed across a snow course and its coincident daily GSv3 SWE estimate. By collecting all data pairs at each snow course location through the period of satellite observations, we can determine how the bias between the GSv3 estimates and reference snow course values behaves with time. The performed analysis^1^ indicated that, in general, the monthly bias observed for a single snow course does not change from year to year, but it varies strongly spatially. This was determined for a time period ranging from 1980 to 2016 across Eurasia and from 1980 to 2003 across North America.

Average bias and its variance is derived for each snow course for each month separately. These biases are spatially interpolated, taking into account the estimated temporal variance, by ordinary kriging interpolation. This results in a monthly hemispheric map of GlobSnow retrieval bias and its spatial variance that are used to bias correct the monthly SWE product. The GSv3 bias correction is time invariant (same correction applied for a given month through the time series), so the technique serves to improve the climatology.

The bias-corrected monthly dataset is generated by applying the bias-correction procedure for each monthly product through the 37 year time series. This is done by subtracting the estimated retrieval-bias on a pixel-level and storing these “bias-corrected SWE estimates” as the bias-corrected monthly products. A fully detailed description is presented in^1^.

## Data Records

### Applied satellite data for SWE retrieval

The satellite PMW data are from the SMMR, SSM/I and SSMIS instruments on board NIMBUS-7 and the Defense Meteorological Satellite Program (DMSP) F-series satellites F8, F11, F13 and F17, respectively. A summary of the PMW sensors are listed on Table 1.

**Table 1:** Summary of three passive microwave sensors applied for SWE retrieval, frequencies relevant for SWE retrieval are listed.

Platform	Sensor	Swath width (km)	Incidence angle (degree)	Total number of frequencies	Frequencies relevant for SWE retrieval (GHz)	Field of view (km)
Nimbus-7 1978–1987	SMMR	800	50.3	5	18.0 37.0	35 × 60 17 × 29
DMSP F8, F11 and F13 1987–2008	SSM/I	1400	53.1	4	19.4 37	45 × 68 24 × 36
DMSP F17 2009->	SSMIS	1700	53.1	21	19.35 37.0	42 × 70 27 × 44

The SMMR was launched in 1978 aboard Nimbus-7 spacecraft and applied for years from 1978 to 1987 with data available every other day^36^.

The SSM/I was the successor to the SMMR instrument. The SSM/I instruments applied for SWE retrieval were onboard DMSP satellites F8, F11 and F13. Data from F8 is applied for years 1987 to 1991, data from F11 is utilized from 1992 to 1995 and data from F13 span 1996 to 2008^37^.

SSMIS followed on from the SSM/I instrument with 24 channels and 21 frequencies^38^. Data from the SSMIS instrument on DMSP F17 is used from 2009 onwards.

The applied SMMR, SSM/I and SSMIS data were acquired from the National Snow and Ice Data Center (NSIDC)^37–39^. Some data gaps exist both in time and space within the satellite passive microwave data record, with coverage generally more consistent from 1987 onwards.

There are differences in local acquisition time between SMMR (noon and midnight equatorial crossing times) and SSM/I (6 am and 6 pm equatorial crossing times, for both SSM/I and SSMIS) which can systematically influence the probability of overpass occurring over a dry versus wet snowpack in ephemeral snow zones and during the melt season^40^. As described in the section on SWE retrieval procedure, the GlobSnow methodology of optimizing an effective snow grain size includes the influence of snow liquid water content, minimizing issues caused by the differences in the timing of satellite observations. The assimilation system also decreases the weight of the satellite data during wet snow periods. These approaches collectively mitigate the SMMR versus SSM/I overpass difference, which is also clear in consistent validation statistics for retrievals from both instruments (presented in the Technical Validation section). The SWE retrieval prioritizes measurements from descending (midnight & 6 pm) crossings, with ascending (noon/6am) data applied only when descending data are not available.

All radiometer observations have been resampled to the EASE-Grid north azimuthal equal-area projection with a nominal resolution of 25 km × 25 km^41^. Resampling the T_B_ measurements to the EASE-Grid allows consistent and co-located time series analysis of the multi-satellite instrument record.

### Weather station snow depth data for SWE retrieval

Weather station SD data are acquired from multiple sources. ECMWF dataset for Eurasia, acquired for years from 1979 to 2018, is complemented by RIHMI-WDC data^42^ which covers the former Soviet Union over the years 1979 to 2018. GHCN-daily SD data^43^ from 1979 to 2018 is used as the main dataset for North America. This dataset is enhanced with data from the Meteorological Service of Canada for years 1979 to 2018 and a large set of measurements from across the continental United States^44^ for latitudes above 40° for years 1979 to 2009.

Each of the five synoptic SD datasets are filtered for duplicate observations. Observations are considered duplicate if the difference between latitude and longitude of the stations is less than 0.001° in which case the median of the observations is used (in case of 2 duplicates, the average is used). After initial filtering, datasets are combined into Eurasian and North American datasets. The two combined datasets are again median filtered for duplicate observations. This time, observations are considered duplicate if they are in the same (25 km × 25 km) EASE-grid pixel, which can occur in regions with a relatively dense surface observing network.

The Eurasian and North American synoptic weather station datasets are then filtered for extremely high SD values (observations over 500 cm are removed). After that, stations with at least 20 measurements for at least 5 separate years are kept, so that stations which report only for brief time periods are removed. Stations where the measured SD is zero for more than 95% of the measurements are also removed. Median filtering is then applied to replace values that differ more than 20 cm from the median value over a 9 day window. Next, stations with unusually deep snow conditions are filtered out, the criteria is met if the mean March SWE exceeds 150 cm in at least 50% of the years that the station has had at least 20 measurements. Lastly, all SD observations above 200 cm are filtered out (i.e. removed).

One further filtering step is conducted for the North American dataset that has a large fraction of 0 cm observations at lower latitudes (the data volume is computationally excessive for the SWE retrieval without this “data reduction” step). For latitudes from 30° to 45°, a 2° by 2° grid is created and the mean of all (filtered) SD observations in each 2° cell is used. For latitude from 45° to 50°, a 1° by 1° grids are used. Data from latitudes above 50° are not reduced through gridding because observations are sparse. Data reduction to 1° and 2° grids for lower latitudes is not applied for Eurasia (reduction is necessary to make the North American dataset computationally feasible).

The initial combined Eurasian dataset has 26 739 497 observations. The final Eurasian dataset after the described filtering steps contains 12 106 721 observations. Initial combined North American dataset has 162 689 571 observations. After the filtering and data reduction steps, the final North American dataset contains 9 638 050 observations. Table 2 shows the number of daily observations in the final Eurasian and North American synoptic weather station SD datasets for winter and summer seasons for different decades. Reasonable consistency is achieved over time.

**Table 2:** Amount of daily observations in the final filtered Eurasian and North American input weather station datasets between 1979–2018.

	Average daily number of records 1979–1989		Average daily number of records 1990–1999		Average daily number of records 2000–2009		Average daily number of records 2010–2018	
	Summer	Winter	Summer	Winter	Summer	Winter	Summer	Winter
All regions	734	748	763	826	680	796	552	849
Eurasia	722	787	774	940	710	943	672	1115
North America	746	709	751	713	649	649	432	583

Winter months cover months between November and April and summer months include months between May and October.

### Reference data for SWE retrieval validation and bias-correction

*In situ* snow course SWE measurements were used for bias-correction of the GSv3.0B monthly product^1^ and to validate the GSv3 and GSv2 daily (Level A) and monthly (Level B) SWE products. Snow course observations consist of manual gravimetric snow measurements made at multiple locations along pre-defined transects several hundreds of metres to several kilometers in length, which are averaged together to obtain a single SWE value for a given transect on a given date. Although sampling protocols vary by jurisdiction, transect-level SWE, depth, and density values, were obtained for Canada^44^, Finland^45^, and Russia^46^. The Canadian network is sampled twice per month (around the 1^st^ and 15^th^) during the snow season while the Finnish network is measured on the 15^th^ of each month with a subset of transects also measured at the end of each month. In Russia, open field sites are measured every 10 days when at least half of the visible area around a station is snow covered; forested sites are sampled once per month prior to 20 January and every ten days thereafter. Sampling frequency of both field and forest sites increases to 5 days during the spring snowmelt season. The Canadian dataset^47^ used for validation extends from 1980–2016, differs slightly from the dataset used for bias-correction^1^ which only covered the 1981–2003 period. The update through 2016 also includes additional sites, particularly in central and northern Quebec.

To mitigate effects of spurious records on the validation results, we applied simple quality checks to the snow course data. For Finland, Russia and Canada, snow transect samples between SWE >0 and < = 500 mm were retained, others discarded (the upper limit was chosen as 500 mm, since the GSv3 product is not provided for mountain regions, where such high SWE values tend occur). For validation, when there was more than one snow transect observation for a given satellite pixel (EASE-grid pixel), the SWE values were averaged together to obtain a more representative value of the landscape SWE. For Canadian data, the quality checks, were applied so that only records with density between 50 and 600 kg m^−3^ and depth >0 and < = 500 cm were retained, following^47^. Records where the re-calculated SWE from depth and density exceeded the recorded SWE by more than 10 mm were also removed, as were those where the re-calculated SWE differed from the reported SWE by more than 10%. This filtering removed ~4% of 1980–2016 records from Canda. A summary of the final filtered snow transect data are shown in Table 3.

**Table 3:** Summary of quality controlled and spatially filtered snow transect data over non-mountain regions.

	All landcover		Boreal Forest		Tundra		Steppe/prairie	
	No. records	Mean SWE [mm]	No. records	Mean SWE [mm]	No. records	Mean SWE [mm]	No. records	Mean SWE [mm]
All regions	603080	83.3	119941	89.2	14903	101.2	102629	63.7
Russia	459535	75.9	94377	81.6	9193	107.9	90876	65.4
Finland	25716	79.6	13525	85.1	495	107.0	0	NA
Canada	117829	112.8	12039	153.2	5215	88.7	11753	50.6

Mountain regions defined by the GSv3 complex-terrain/mountain mask (section on Input Data).

### The GSv3 daily, monthly and monthly bias-corrected datasets

The monthly GSv3 data record^48^ is available via the PANGAEA: 10.1594/PANGAEA.911944.

The daily GSv3 data record is available at: https://www.globsnow.info/swe/archive_v3.0/.

## Technical Validation

### Assessment of the SWE retrieval accuracy for the GS v3.0 product

**Fig. 3:**
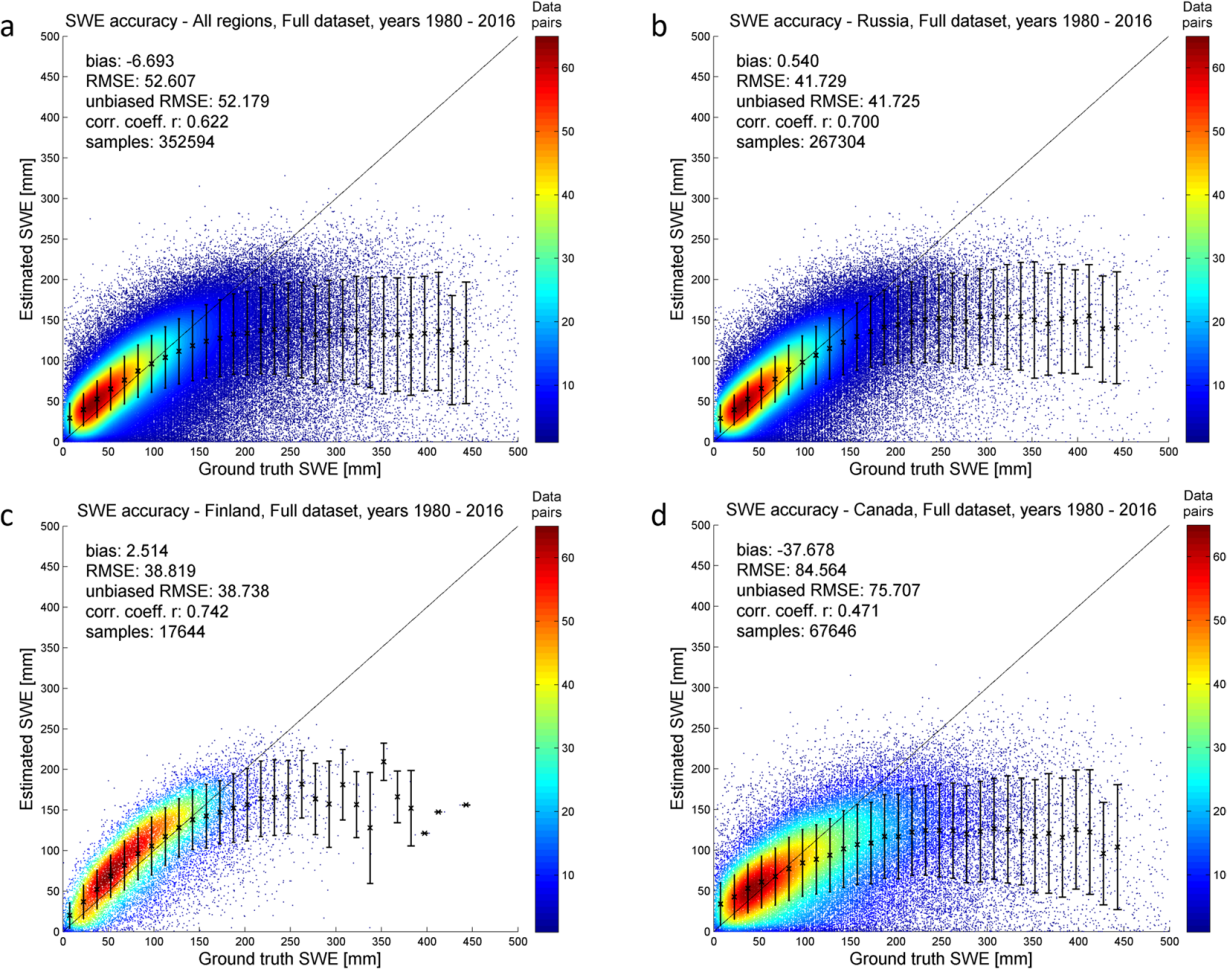
Scatter plot of GSv3 retrieval accuracy for 1980–2016. (Panel a) shows results for all samples and all regions; (panel b) shows results for Russia; (panel c) shows results for Finland; (panel d) shows results for Canada.

The GSv3 daily SWE product retrievals were matched with co-located snow transect SWE observations for 1980–2016 (01/1980–12/2016) and bias, rmse, and correlation statistics were computed (Tables 4 and 5). Assessment was made for all samples (Table 4) and for samples where snow transect data indicated shallow to moderate snowpack (SWE below 150 mm) (Table 5). The overall RMSE for 1980–2016 is 52.6 mm. GSv3 slightly overestimates SWE in Eurasia and underestimates SWE across Canada (note the mean snow transect SWE is considerably greater for Canada than Russia). The overall RMSE for shallow to moderate snow conditions (SWE below 150 mm) is 32.7 mm, which indicates a notably better accuracy, and emphasizes the challenge for the satellite-based GlobSnow approach to estimate deep snow conditions as noted in^49^ and in previous GlobSnow versions (e.g.^3^). The results represent an update from the performance reported earlier^1^ because of the additional Canadian snow course reference data covering 2004–2016 and additional locations covering 1979–2003 included in these results. Retrieval accuracy and number of data pairs (reference vs estimate) for different ranges of estimated and reference SWE are illustrated in Fig. 3 (panels a-d) for different regions. The plots indicate generally a very good retrieval performance up to 150 mm SWE ranges. Deeper snow conditions are typically underestimated. The great majority of the data pairs are for SWE conditions between 0 mm and 150 mm, which indicates strong algorithm performance across the most typical SWE values.

**Table 4:** Number of data pairs, bias, RMSE, correlation, for GSv3.0 against snow course transects from Canada, Finland, and Russia; mean SWE as measured from snow transects.

	Number of data pairs	Bias [mm]	RMSE [mm]	Correlation (r)	Mean SWE [mm]
All regions	352594	−6.7	52.6	0.62	89.0
Russia	267304	0.5	41.7	0.70	79.9
Finland	17644	2.5	38.8	0.74	93.9
Canada	67646	−37.7	84.6	0.47	123.7

Results for all samples and all months 01/1980–12/2016.

**Table 5:** Number of data pairs, bias, RMSE, correlation, for GSv3.0 SWE data below 150 mm against snow course transects from Canada, Finland, and Russia.

	Number of data pairs	Bias [mm]	RMSE [mm]	Correlation (r)
All regions	298138	6.4	32.7	0.66
Russia	240041	8.2	30.5	0.70
Finland	14839	10.0	32.4	0.74
Canada	45597	−4.3	42.5	0.46

Results for all samples, where snow transect indicates SWE <150 mm and all months 01/1980–12/2016.

**Fig. 4:**
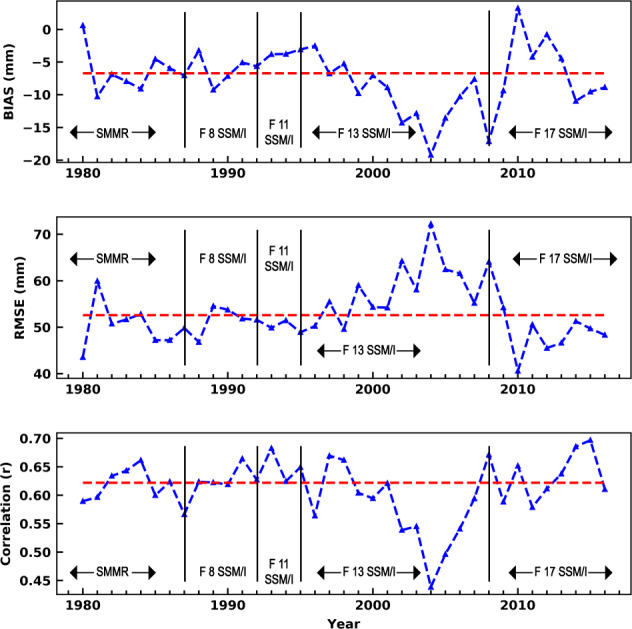
GSv3 bias, RMSE, correlation for each year between 1 January 1980 and 31 December 2016 (blue); 37 year mean (red).

**Fig. 5:**
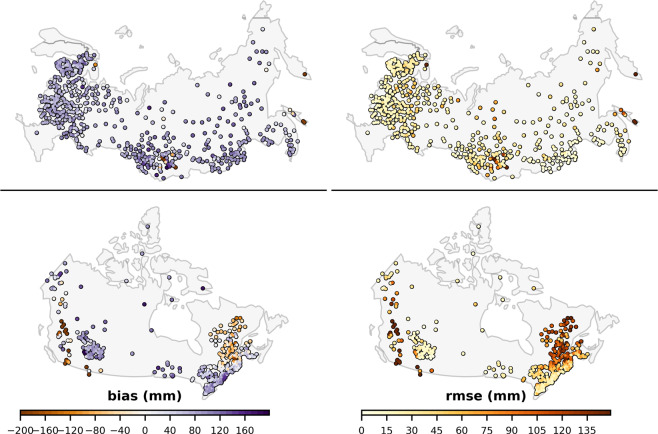
Bias and RMSE for all March GSv3-snow course data pairs within 25 km EASE grid cells for the period 1980–2016. Grid cells with 10 or fewer data pairs and data pairs in five or fewer different years are not shown. Bias and RMSE limits set to ±200 mm and 150 mm, respectively, for display purposes.

**Fig. 6:**
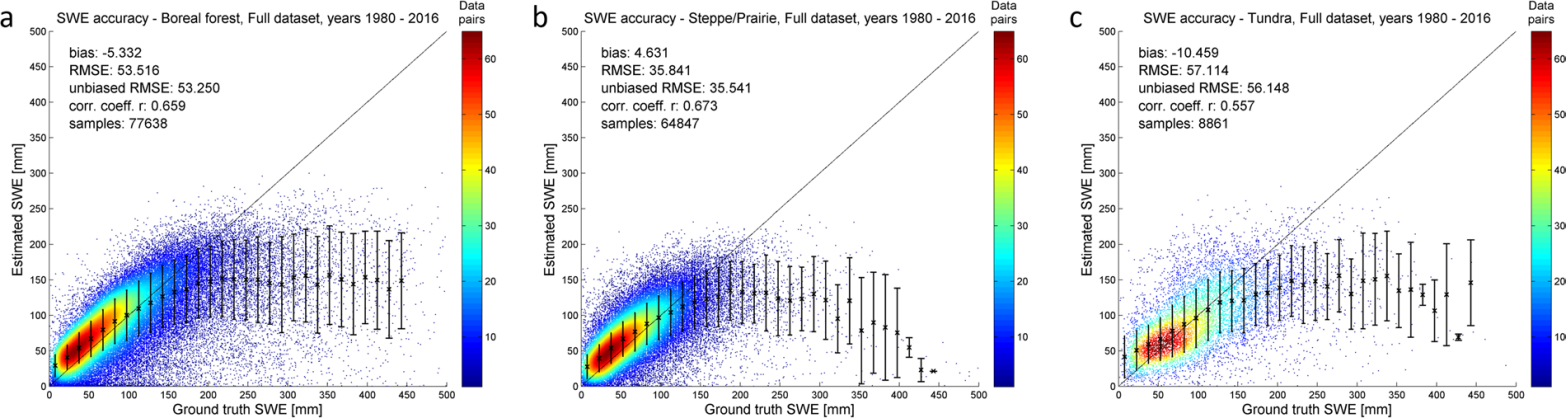
Reference SWE (snow transect) versus estimated SWE (GSv3 daily) for data pairs located in boreal forest (panel a), steppe/prairie/agricultural (panel b) and tundra landcover zones (panel c). ESA GlobCover classification is used to determine the landcover zones.

**Fig. 7:**
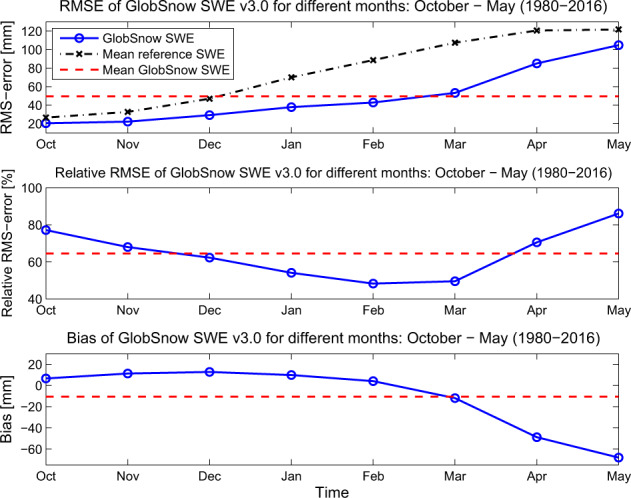
RMSE, Relative RMSE and Bias for GSv3 daily SWE data for different months. Mean reference SWE is the snow transect based average SWE for the given month. Relative RMSE is the RMSE given in percentage, when compared with the mean reference SWE for the given month.

Figure 4 presents the retrieval accuracy assessed independently for different years. There is no apparent trend in bias, RMSE, or correlation over the 37 year validation period (Fig. 4). Encouragingly, the figure highlights similar retrieval performance during the SMMR-era when compared with the performance from SSM/I and SSMIS sensors. The retrieval performance is presented for different geographical regions in Fig. 5. Locations with a high RMSE tend to have large negative biases (north-east and western Canada, high elevation regions of central Eurasia, the pacific coast of Eurasia) and often coincide with areas of high SWE. This is consistent with the degradation in product performance when reference SWE is above ~150 mm, especially in the boreal forest and tundra regions (Figs. 5 and 6). The SWE retrieval performance is assessed for different seasons by calculating the RMSE and bias separately for each month, shown in Fig. 7. These results show that although the absolute RMSE is lower during the accumulation season (October – December) the RMSE relative to the the mean reference SWE is higher compared to mid-winter (January, February and March) when the relative RMSE is at a minimum and SWE is at a maximum.

The largest RMSE is observed in April and May, most likely due to deeper snow packs (in high latitudes) and uncertainty due to masking wet snow cases during the snow melt season. The observed bias tends to be positive during the early winter season (with shallow snow packs) and negative later in the season due to deeper snow conditions. The utilization of constant snow density in the SWE retrieval methodology explains some of the bias behaviour between early and late season, as snow density is typically lower during the early season and higher in late spring relative to the constant density applied in the GSv3 retrieval.

### Comparison between GSv2, GSv3 and the GSv3 bias-corrected data

The performance of GSv2^3^ and GSv3 datasets relative to the reference SWE measurements is presented in Table 6. There is notable improvement in RMSE and correlation for the Russian domain from GSv2 to GSv3, no measureable change in retrieval performance over Finland, and a small decrease in accuracy over the Canadian domain. Overall, the retrieval performance is improved over GSv2 with a smaller bias, a lower RMSE, and a stronger correlation for all regions combined.

**Table 6:** Number of data pairs, bias, RMSE, correlation, for GSv2.0 and GSv3.0 SWE against snow course transects from Canada, Finland, and Russia.

	Number of data pairs	Bias [mm]	RMSE [mm]	Correlation (r)
All regions - GSv2.0	343070	−6.5	53.8	0.60
All regions - GSv3.0		−5.9	51.5	0.63
Russia - GSv2.0	260285	−0.1	45.2	0.64
Russia - GSv3.0		1.2	40.8	0.71
Finland - GSv2.0	16985	4.0	38.7	0.75
Finland - GSv3.0		3.2	38.5	0.75
Canada - GSv2.0	65800	−34.5	81.3	0.51
Canada - GSv3.0		−36.5	82.9	0.48

Results for all samples and all months 01/1980–12/2016 (only data points that are available on both products were used).

Assessment of the GSV3B bias-corrected product for March 1980–2016 is challenging because the same snow transect data are used for validation and bias correction. The bias-correction field for each month is calculated from the full snow course data record, spanning all years and all samples for the given month to produce a time-invariant average over the whole 37 year record. The same monthly bias correction is applied through the entire time series (e.g. the same bias correction for March is applied every year through the time series), thus individual *in-situ* samples are not fully correlated with the bias-corrected SWE estimates but are also not fully independent. For this reason, a rigorous assessment cannot be performed using the currently available reference data and only an approximate assessment of retrieval performance for bias corrected March SWE is feasible^1^.

Bias of the uncorrected data is roughly equal to the bias-corrected data less the value of the correction field at the points sampled. Interannual variability in the residual bias (after correction) through the 37 year record approximately follows the annual SWE anomalies such that anomalously high SWE years still contain a negative bias (under-correction) while anomalously low SWE years retain a positive bias (over-correction). Given this lack of independence, validation statistics were not produced for the bias-corrected product.

The impact of the bias correction procedure is to a degree dependent on the density of available weather station observation network. In general, the higher the density of *in situ* SD observations the more accurate is the SWE retrieval and the associated bias correction is therefore smaller. For Finland, the bias correction impact is minimal because the country is covered by a dense network of weather stations that provide comprehensive inputs of snow depth to the initial retrieval, hence algorithm performance is generally strong (Fig. 3c; Table 2). As available stations decrease (across Russia and Canada) and the typical snowpack becomes deeper (particularly notable across eastern parts of Canada), the bias correction becomes more pronounced.

Additionally, the density of snow course network used for the bias-correction affects the accuracy of bias correction. Kriging interpolation provides an estimate of the uncertainty of estimated bias for different grid cells. Further, the level of maximum error of regional or hemispheric bias-corrected snow mass estimates can be obtained by leave-one-out analysis of applied regional snow course data set. In practice, the reliability of regional snow mass estimates increases with the increasing size of region of interest.

### GS v3.0 SWE retrieval performance in relation to distance from weather stations

The impact of distance from the weather stations reporting SD on GSv3 retrieval performance was assessed for March 1980–2018. The distance from snow course measurements to the nearest reporting weather station for all co-located March snow course – GSv3 data pairs was calculated. The performance of the GSv3 SWE retrieval versus direct interpolation of weather station SD data (where SWE is obtained from SD by applying a constant snow density of 0.24 g cm^−3^) was also assessed to quantify improvements obtained by fusing satellite data with the background SD field.

**Fig. 8:**
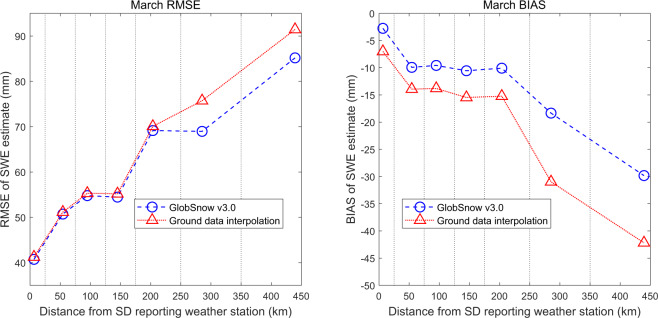
Observed RMSE and SWE retrieval bias as a function of the distance from the nearest weather station reporting SD, northern hemisphere in March. In addition to GSv3 SWE the results obtained by ground data (kriging) interpolation are shown.

The GSv3 SWE retrieval performance decreases as a function of the distance from the SD reporting weather stations. As shown in Fig. 8 for the northern hemisphere, both the bias and RMSE deteriorate significantly with increasing distance. Interestingly, the retrieval bias is better for GSv3 compared to direct interpolation of synoptic weather station SD even for short distances, i.e. for data points in regions with a dense weather station observations network. This is an important result and confirms that improvement are gained from fusing space-borne passive microwave brightness temperatures with *in situ* snow depth data^2,3^. In terms of RMSE, the quantitative improvement obtained by fusing the satellite data becomes more important at distances over 200 km. Overall, the (March) RMSE is better for GSv3 retrievals (57.8 mm) compared with direct interpolation of weather station SD (59.4 mm).

## Known Limitations

GSv3 data are produced and available for non-mountain regions of the Northern Hemisphere. Because of known limitations in complex-terrain, a mask is applied based on the sub-grid variability in elevation determined from ETOPO5^21^ data. All land ice and large lakes are also masked; retrievals are not produced for coastal regions of Greenland.

The algorithm is best used to measure snow packs roughly between 0.05 m and 1.00 m in depth. Depths less than 0.05 m will not be reliably retrieved because passive microwave sensitivity under very shallow snow conditions falls within the 2 K detection precision of the radiometer instruments used. With SWE greater than 150 mm (typically occurring at snow depths greater than 1 m), the brightness temperature signal starts to saturate causing underestimation of SWE even though the weight of satellite-derived information drops compared with the weight of *in situ* snow depth observations in the assimilation algorithm. Even a relatively small amount of liquid water in the snowpack reduces the sensitivity of radiometer observations to SWE. Consequently, the weight given to the satellite data drops towards zero in the assimilation algorithm under wet snow conditions. SWE estimates for fully wet snow conditions are derived entirely from the weather station kriged background SD field the accuracy of which is affected by the density of, and distance from, nearby weather stations.

The GSv3 product is also dependent on the spatial density of the weather station SD observations available to calculate the background snow depth and snow grain size fields that are applied in the SWE retrieval. Where the surface network is sparse, the SWE estimates are less accurate, especially in regions where large variations in SWE level are typical (e.g. across tundra environments) and pointwise measurements are unable to describe the range in SWE conditions. It should be noted, however, that while the absolute uncertainty of the retrieval may be higher in surface data sparse regions, the assimilation algorithm does provide value-added information compared to simply kriging the surface SD observations, especially at longer distances (Fig. 8).

The daily uncertainty layer provided with the GSv3 product provides information on the reliability of the SWE retrieval for the given pixel determined through the statistical standard deviation of the SWE estimates. If a user has a known threshold for the retrieval accuracy required by their respective end-user applications the SWE uncertainty field can be applied as a data usability flag.

Beside limitations caused by signal saturation and availability of weather station data, the daily product is subject to biases caused by using a static snow density value, a necessary simplification at the present time. The density of dry snow usually varies between 0.100 g cm^−3^ (fresh, recently fallen snow) and 0.400 g cm^−3^ (compacted and wind-influenced snow). The applied value of 0.240 g cm^−3^ therefore provides reasonable results at the continental scale and across the entire snow season but results in a seasonal bias that users should be aware of. As a result SWE values tend to be somewhat overestimated during the early accumulation period and underestimated during the late winter and spring period. The bias-correction procedure mitigates this issue for the monthly bias-corrected product version.

## Usage Notes

The product data are available from: “https://www.globsnow.info/swe/archive_v3.0/”, and 10.1594/PANGAEA.911944.

The daily SWE product for 01/1979–09/1987 is available for every second day. The SWE product for 09/1987–2018 are available for every day during winter season (when enough seasonal snow is available for retrival). Due to anomalies and inconsistencies in ground-based weather station snow record and gaps in the satellite passive microwave data, there are some periods for which the SWE data are unavailable.

Sufficient snow transect data for bias-correction are available from January to May; thus the bias-corrected monthly data are currently not available for months of June to December.

The GlobSnow retrieval framework was implemented and the development is continuing as part of the European Space Agency Climate Change Initiative – Snow (ESA CCI Snow) project. The initial Snow CCI SWE dataset release follows the processing chain as outlined in Fig. 1, but with some small differences in the input snow depth data from weather stations, and the final output product grid format. These data are available via the ESA CCI data portal (climate.esa.int/data). Subsequent algorithm enhancements will be implemented in future releases of the Snow CCI SWE product.

## Data Availability

The codes are available from: https://github.com/fmidev/GlobSnow3.0. and also from: http://www.globsnow.info/swe/archive_v3.0/source_codes/.
